# Understanding plant development for plant breeding

**DOI:** 10.1270/jsbbs.73.1

**Published:** 2023-03

**Authors:** Jun-ichi Itoh, Yutaka Sato

**Affiliations:** 1 Graduate School of Agriculture and Life Sciences, The University of Tokyo; 2 Genome and Evolutionary Biology, National Institute of Genetics; 3 Department of Genetics, School of Life Science, SOKENDAI (The Graduate University for Advanced Studies)

Morphological traits have been the most important targets for plant improvement in the long history of crop breeding, including the domestication of crops that occurred more than 10,000 years ago and current cutting-edge molecular breeding. Modification of morphological traits has improved various agronomically important traits in ancestral varieties and contributed to the breeding of high-yielding, agronomically superior varieties. Agronomic productivity is not solely determined by the final morphology of a crop. From sowing to harvest, the farmer’s care and management affect the growth and development of crops, and consequently productivity. In addition, it is crucial to understand how crop morphology changes in response to the environment and conditions. Therefore, crop developmental biology has been an important research area not only for curiosity-driven biological science but also for plant breeding and agriculture.

In the history of plant developmental biology, the promotion of molecular genetics in the 1990s and the genome decoding of higher plants beginning in 2000 had a great impact on the direction of research. In 2000, the whole genome sequence of *Arabidopsis* was first published as a model of higher plants, and that of rice was released in 2004 as a model crop. These led to the elucidation of the molecular entity of the semi-dwarf genes used in the Green Revolution (Hedden 2003), the identification of genes that contributed to the domestication of various crops (Doebley *et al.* 2006), and the isolation of Mendelian genes (Ross and Reid 2011). Subsequently, with the development of quantitative trait locus (QTL) analysis and genome-wide association studies (GWAS), the genes affecting various agronomic morphological traits have been isolated and analyzed (Meyer and Purugganan 2013). Although such studies have not always been conducted from a developmental perspective, further analyses have strengthened our understanding of the genetic and biological functions of the genes involved in plant development. Conversely, studies of genes involved in important developmental processes in model plants have demonstrated their potential for improving new agronomic traits in crops (Li *et al.* 2018).

Advances in plant developmental biology and genetics have relied on a few model plants with well-annotated genome information, genetic resources, and molecular tools. However, the important questions regarding plant development and useful agronomic traits are often associated with morphological characters that are unique to crops. Therefore, it is extremely important to understand the mechanisms of morphological traits that do not exist in model plants and to use them. Recent advances in genome technologies and informatics make molecular approaches more accessible for important non-model crops, which will make it possible to unveil the genetic and molecular mechanisms of morphological traits that are characteristic to individual crops. Furthermore, combined with genome-editing technologies, findings regarding the developmental processes of crops should become even more important for molecular breeding (Hua *et al.* 2019, Rodríguez-Leal *et al.* 2017).

This special issue of *Breeding Science* contains five reviews and one research paper that focuses on recent advances and current challenges in developmental research in crops. These articles cover the developmental biology of rice (Tanaka *et al.* 2023), wheat/barley (Sakuma and Koppolu 2023), sorghum (Takanashi 2023), legumes (Suzaki 2023), and tomato (Nakayama *et al.* 2023), as well as rice panicle architecture (Agata *et al.* 2023). All of these papers cover research focused on the unique characteristics of the specific crop, and they suggest the importance of developmental studies that support the foundation of plant breeding and give us different perspectives from that of model plants such as *Arabidopsis*.

We thank the authors of the articles featured in this special issue for their contributions and thoughtful insights into current advances in this research field. These articles provide valuable information that can be used in future developmental studies and crop breeding. Finally, we would like to thank the Editor-in-Chief, Dr. Tsujimoto, for giving us the opportunity to edit this special issue.
